# Fructose in Breast Milk Is Positively Associated with Infant Body Composition at 6 Months of Age

**DOI:** 10.3390/nu9020146

**Published:** 2017-02-16

**Authors:** Michael I. Goran, Ashley A. Martin, Tanya L. Alderete, Hideji Fujiwara, David A. Fields

**Affiliations:** 1 Department of Preventive Medicine, University of Southern California, 2250 Alcazar Street, CSC 200, Los Angeles, CA 90033, USA; ashley.ann.martin@gmail.com (A.A.M.); tanya.alderete@usc.edu (T.L.A.); 2 School of Medicine, Washington University in St. Louis, St. Louis, MO 63110, USA; hideji.fujiwara@gmail.com; 3 Department of Pediatrics, University of Oklahoma Health Sciences Center, Oklahoma City, OK 73104, USA; David-Fields@ouhsc.edu

**Keywords:** breastfeeding, breast milk, maternal programming, added sugars, fructose

## Abstract

Dietary sugars have been shown to promote excess adiposity among children and adults; however, no study has examined fructose in human milk and its effects on body composition during infancy. Twenty-five mother–infant dyads attended clinical visits to the Oklahoma Health Sciences Center at 1 and 6 months of infant age. Infants were exclusively breastfed for 6 months and sugars in breast milk (i.e., fructose, glucose, lactose) were measured by Liquid chromatography-mass spectrometry (LC-MS/MS) and glucose oxidase. Infant body composition was assessed using dual-energy X-ray absorptiometry at 1 and 6 months. Multiple linear regression was used to examine associations between breast milk sugars and infant body composition at 6 months of age. Fructose, glucose, and lactose were present in breast milk and stable across visits (means = 6.7 μg/mL, 255.2 μg/mL, and 7.6 g/dL, respectively). Despite its very low concentration, fructose was the only sugar significantly associated with infant body composition. A 1-μg/mL higher breast milk fructose was associated with a 257 g higher body weight (*p* = 0.02), 170 g higher lean mass (*p* = 0.01), 131 g higher fat mass (*p* = 0.05), and 5 g higher bone mineral content (*p* = 0.03). In conclusion, fructose is detectable in human breast milk and is positively associated with all components of body composition at 6 months of age.

## 1. Introduction

Added sugar is an established risk factor for obesity as well as related metabolic diseases including type 2 diabetes, cardiovascular disease and non-alcoholic fatty liver [1,2,3,4,5]. Fructose appears to be at least partially responsible for this detrimental relationship, as fructose metabolism is unregulated [6,7] and fructose has been linked to greater adiposity and metabolic disturbances compared to other sugars that appear to be especially important during critical periods of growth and development [8]. While many studies have examined the impact of fructose and other sugars on body weight in childhood, few studies have examined the impact of early exposures to these sugars during infancy [9,10].

One of the most direct routes by which infants may be exposed to fructose and other sugars is through breastfeeding. During the first six months of life, many infants obtain their nourishment predominately if not exclusively from breast milk, which contains a variety of macronutrients and other relevant factors (e.g., cytokines, appetite hormones) [11,12,13]. While this exclusive breastfeeding period would appear to preclude any access to fructose exposure in early-life, studies have shown that the composition of breast milk is shaped by the maternal diet [14,15]. Therefore, breast milk may contain varying levels of maternal dietary macronutrients that have the potential to contribute to childhood obesity and future metabolic disease risk [16,17,18,19]. For instance, in infants born to mothers diagnosed with gestational diabetes (GDM), consuming greater volumes of ‘diabetic’ milk in the first week of life has been found to be associated with a 2-fold increased risk of being overweight at age 2 [20]. This effect is attributed, in part, to the higher levels of glucose and insulin observed in the breast milk of mothers with GDM [21,22]. Breast milk concentrations of glucose and insulin have also been found to positively predict adiposity in infants born to non-diabetic mothers [12]. Recent research suggests that even non-nutritive carbohydrates found in breast milk (i.e., human milk oligosaccharides) have the potential to contribute to infant growth and body composition in infancy [23]. Together, these studies highlight the need to better understand the composition of breast milk and whether macronutrient composition affects infant growth and development.

The first aim of this study was to determine whether fructose was detectable in human breast milk. The second aim was to examine if fructose in breast milk was associated with infant weight and body composition at six months of age. We tested this hypothesis within a cohort of 25 mother-infant pairs where infants were exclusively breastfed, analyzing relationships between breast milk sugar composition and infant growth and body composition. To our knowledge, this study is the first to examine the associations between breast milk fructose and infant body composition.

## 2. Methods

### 2.1. Study Overview

Thirty-seven mother-infant dyads were enrolled at 1-month (±5 days) of age for participation in a 6-month longitudinal exclusively breastfeeding growth study. Of the 37 mother-infant pairs initially enrolled in this study, 25 were retained in this analysis. Eleven participants were excluded due to loss of follow-up for milk analyses (either due to dropping out or declining to produce a milk sample) and another participant was excluded because they did not produce enough breast milk for analysis. The overall study design and preliminary results were described in a previous study that examined the relationships between breast milk hormones and inflammatory markers [12]. The data reported here describe relationships between infant body composition and breast milk sugar content that were not explored in the previous work. In brief, participants were instructed to arrive at the University of Oklahoma approximately between 8:00 a.m. and 10:00 a.m. with the mother fasted at least 3 h. Upon arrival, a single breast-milk expression was obtained from the mother with a whole-body dual energy X-ray absorptiometry (DXA) scan conducted on the infant. Measurements were performed when the infant was 1 month of age (used as baseline covariates in this analysis) and were repeated at 6-months of age (all visits were ± 5 days). All subjects gave their informed consent for inclusion prior to participating in the study. The study was conducted in accordance with the Declaration of Helsinki, and the protocol was approved by the Ethics Committee of the University of Oklahoma Health Sciences Center (IRB #4426 and 5297).

### 2.2. Participants

The 25 mother-infant dyads were exclusively breast-fed (defined <8 ounces of formula a week though no subject consumed any formula past 7 days of age) with the following inclusion criteria used: (a) maternal age at delivery between 18 and 45 years old; (b) gestation ≥37 weeks; (c) singleton birth; and (d) a postpartum hospital stay for mother and infant less than 3 days. Participants were excluded for any of the following: (a) maternal use of tobacco; (b) mothers’ alcohol consumption exceeding one drink per week; (c) type 1 or 2 diabetes prior to or during pregnancy; or (d) the infant was born with presumed or known congenital birth defects. All maternal demographic information (age, parity, pre-pregnancy weight and gestational weight gain) was collected by medical chart abstraction when possible.

### 2.3. Anthropometric and Body Composition Variables

Crown-to-heel length was measured in duplicate using a Seca 416 infantometer (Seca, Hamburg, Germany) with both measures being within 0.1 cm. Nude body weight was measured in duplicate with a Seca 728 scale (Seca, Hamburg, Germany) with both measures being within 10 g. On the rare occasion these measures exceeded the criteria set forth, a third measure was obtained and the two closest values averaged. Weight-for-length z-scores were calculated using World Health Organization data. Total adiposity (% fat), fat mass (g), lean mass (g), bone mineral density (g/cm^2^), and bone mineral content (g) were collected using a Lunar iDXA (General Electric, Fairfield, CT, USA) scanner as described previously [12]. During the scan, the infant wore only a diaper and was swaddled in a light blanket. The principal investigator (DAF) positioned all infants and performed subsequent scan analyses.

### 2.4. Breast-Milk Collection

Mothers came to the University of Oklahoma Health Sciences center for their study visits typically arriving between 8:00 a.m. and 10:00 a.m. The laboratory provided a hospital-grade breast pump for pumping (Symphony^®^ Breast Pump, Medela Inc., McHenry, IL, USA) while the mother was encouraged to completely empty the right breast for the quantification of breast milk sugars. While mothers were encouraged to pump from the right breast for uniformity, each mother was allowed to decide from which breast she pumped.

### 2.5. Breast-Milk Analyses

#### Preparation of Fructose Quantification Sample

The protein was removed by precipitating from the breast milk (50 µL) with addition of 200 µL of acetonitrile containing 10 µg of carbon-13 labeled (^13^C_6_)-fructose as the internal standard for natural fructose quantification in the breast milk. The supernatant, which contained natural fructose as well as ^13^C_6_-fructose, was collected after centrifugation of the protein precipitated breast milk for MS analysis. The 5-point calibration samples were prepared for absolute quantification of fructose in the breast milk. QC (quality control) samples were also prepared from pooling some of individual supernatants for monitoring on analytical performance throughout fructose analysis.

Preparation of lactose quantification sample: The original breast milk was initially diluted by 100-fold with water. Then, 5 µL of the diluted breast milk was again diluted by 200-fold with 995 µL of water, containing 5 µg of ^13^C_12_-lactulose as the internal standard. The 4-point calibration samples were prepared for absolute quantification of lactose in the breast milk. QC samples were prepared for lactose analysis.

LC-MS/MS Analyses: The fructose and lactose analyses were performed with a Shimadzu 20AD HPLC system (Shimadzu USA, Columbia, MD, USA) and a Leap PAL (Palparts) autosampler (Leap Technologies, Carrboro, NC, USA) coupled to a triple quadrupole mass spectrometer (API (Atmospheric Pressure Ionization) -4000: Applied Biosystems, Foster City, CA, USA). Initially, 3 µL of fructose and lactose samples were separately injected onto an Imtakt UK-amino HPLC column (3 × 100 mm, 3 µm: Imtakt USA, Portland, OR, USA) with the following mobile phases: (a) 10 m mole ammonium acetate in water; (b) acetonitrile at a flow rate of 1 mL/min. The column was heated at 60 °C throughout the analyses. The positive ion ESI MRM mode was used for detection of fructose (Q1/Q3:198/145) and lactose (Q1/Q3:360/163) as well as ^13^C_6_ fructose (Q1/Q3:204/151) and ^13^C_12_-lactulose (Q1/Q3:372/169). Data processing was conducted with Analyst 1.5.1 (Applied Biosystems, Foster City, CA, USA). Unlike GC-MS (Gas Chromotrography-Mass Spectrometry) quantification of fructose [24] that can sometimes overestimate concentrations due to incomplete derivatization (see Supplementary Materials Figure S1), our LC-MS/MS method does not require the derivatization of fructose and lactose at all and, thus, is not subject to these concerns.

Glucose and insulin quantification: Milk fat was separated from the aqueous phase by centrifugation with the resulting skimmed milk assayed using commercially available immunoassay kits for insulin described by our group previously [12]. Glucose was measured using the glucose oxidase method (2300 STAT Plus, Yellow Springs Instruments, YSI Incorporated, Yellow Spring, OH, USA).

### 2.6. Statistical Analysis

Paired *t*-tests were used to assess the change in infant growth and breast milk sugar content from 1 to 6 months. These *t*-tests indicated that sugar and insulin levels in the milk did not significantly vary between 1 and 6 months; thus, all statistical models were conducted using the average of these two values (e.g., average glucose, fructose, lactose, and insulin).

To examine how breast milk levels of fructose, glucose, lactose, and insulin relate to infant growth, separate hierarchical regression models were conducted predicting infant weight, length, weight-for-length z-score, fat mass, lean mass, bone mineral density (BMD), bone mineral content (BMC), and overall percent adiposity at 6 months of age. A priori covariates included mothers’ pre-pregnancy body mass index (BMI), 1-month infant weight (g), and infant sex. Pearson correlation coefficients conducted during data exploration indicated that 1-month weight was highly correlated with 1-month length (*r* = 0.71), 1-month fat mass (*r* = 0.90), 1-month fat free mass (*r* = 0.95), and 1-month overall adiposity (*r* = 0.68). Thus, for simplicity and to avoid multicollinearity among predictors, only 1-month weight was retained in the base model. Thus, Step 1 of the hierarchical model consisted of our base model (infant sex, infant 1-month weight, mother prepregnancy BMI) and Step 2 introduced the particular milk component of interest (e.g., average fructose, glucose, lactose, insulin concentration). This approach allowed us to examine the contribution of milk sugar content towards explaining the variance in infant growth after controlling for known predictors of infant weight outcomes. Results are presented as the mean ± standard deviation (M ± SD). Unless otherwise stated, models were conducted with the continuous predictor variables centered on the mean; unstandardized beta coefficients (β) are reported. All assumptions of multiple linear regression were satisfied. Analyses were performed in SPSS version 22 with a priori significance level set at *p* < 0.05 (SPSS, IBM, Armonk, NY, USA).

## 3. Results

### 3.1. Variation in Breast Milk Sugar Composition within the First Six Months of Life

**Figure 1:**
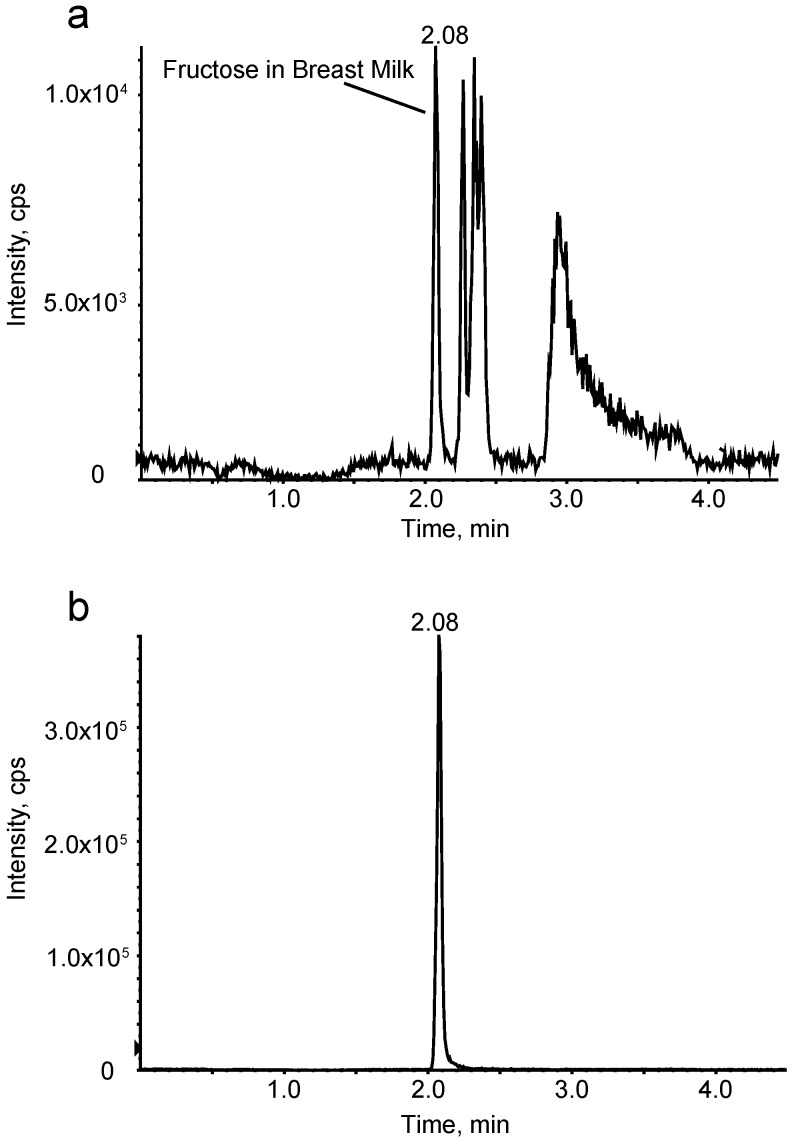
LC-MS/MS (Liquid Chromatography-Mass Spectrometry/Mass Spectrometry) chromatographic traces confirming fructose content in breast milk. (**a**) Natural fructose in breast milk (sample # 108-1); (**b**) Spiked Carbon-13 labeled ^13^C_6_-fructose as the internal standard (10 μg in 50 μL of breast milk).

The characteristics of the 25 mother–infant pairs enrolled in the study are shown in Table 1. As expected, infants’ weight, length, lean mass, fat mass, and overall adiposity significantly increased over the six-month study period (*p* < 0.001). The milk sugar concentration at both one and six months are reported in Table 2. As shown in Figure 1, we were able to accurately measure fructose content in breast milk using LC-MS/MS, displayed as typical chromatographic traces. Milk sugar remained constant over time, as indicated by non-significant *t*-tests comparing one and six months.

### 3.2. Relationships between Breast Milk Sugar Content and Infant Growth

**Figure 2:**
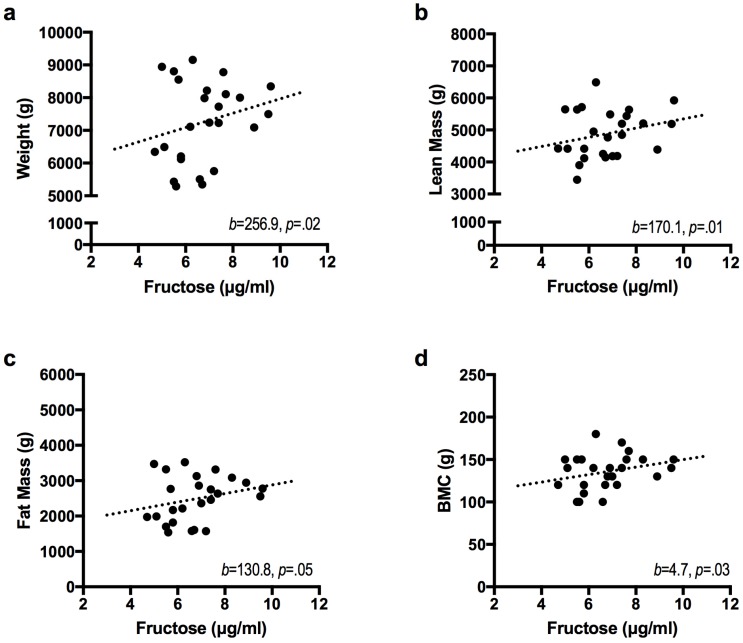
Breast milk fructose is positively associated with infant body composition at 6 months of age. Figures display the unadjusted values for breast milk fructose relative to (**a**) body weight; (**b**) lean mass; (**c**) fat mass; and (**d**) bone mineral content. Hierarchical linear regression was performed to obtain the parameter estimates (*b*) after adjusting for infant sex, one-month infant weight, and maternal body mass index (*n* = 25).

Given that the breast milk concentrations of each sugar did not vary between one and six months, the average concentration of each sugar was calculated and entered into separate hierarchical regression models predicting infant body composition at 6 months of infant age. These results are displayed in Table 3. Even after adjusting for baseline covariates (ome-month infant weight, infant sex, and maternal BMI), breast milk fructose accounted for an additional 8% of the variance in infant weight (*p* = 0.02), an additional 9% of the variance in infant lean mass (*p* = 0.01), an additional 7% of the variance in infant fat mass (*p* = 0.05), and an additional 9% of the variance in infant bone mineral content (*p* = 0.03) at 6 months of age. As shown in Figure 2, each 1-μg/mL increase in fructose was associated with a 257 g increase in body weight (β = 256.9, *p* = 0.02), 170 g increase in lean mass (β = 170.1, *p* = 0.01), 131 g increase in fat mass (β = 130.8, *p* = 0.05), and 5 g increase in bone mineral content (β = 4.7, *p* = 0.03). A positive relationship was also observed for fructose predicting increased weight-for-length z-scores (β = 0.3, *p* = 0.02). There was no evidence that infant growth was related to mothers’ pre-pregnancy BMI (largest *p* = 0.59; from base model) or to any of the other breast milk components.

## 4. Discussion

The detrimental effects of fructose are well documented in children and adults, but no study that we are aware of has examined whether fructose is detectable in human breast milk, and whether this might be associated with growth and body composition, particularly adiposity in infancy. These associations were observed even though the level of fructose in breast milk was extremely low (7 μg/mL), approximately 1/30th the level of glucose. Despite this very low concentration, fructose levels in breastmilk appeared to be biologically relevant. Each 1-μg/mL higher fructose was associated with a 257 g higher body weight, 170 g higher lean mass, 131 g higher fat mass, and 5 g higher bone mineral content at six months of age in our sample. These effects remained significant even after accounting for covariates known to impact infant growth, such as sex, baseline weight, and maternal BMI prior to pregnancy and were not apparent for glucose levels in breastmilk.

In this small proof of concept study, we observed that fructose was significantly associated with higher body weight, and that this effect was distributed across all components of body composition (i.e., fat mass, lean mass, and bone mass). It is important to note that these observed associations do not necessarily imply causation. Further work is needed to examine the possibility that even these very small amounts of fructose can affect musculoskeletal development as well as adipose tissue development in infancy and early life, which is a rapid growth period where significant changes in muscular and skeletal growth are occurring. Indeed, although evidence investigating age-related changes are generally lacking in this area, our results are consistent with a recent paper which found that a high fructose diet led to increased skeletal density and increased length in adolescent rats [25]. Further research is needed examining the effects of fructose in early life, including at low doses, in order to draw more causative conclusions about the specific impact of fructose on infant adiposity and tissue development.

Since human milk does not naturally contain fructose [26], our findings highlight maternal intake of fructose-containing products, such as sugar sweetened beverages, as a targetable intervention for reducing exposure to fructose in early life. While previous studies have shown that fructose can be transmitted in utero through the placenta [27,28], our findings extend this literature by identifying breast milk as a potential route of fructose transmission in the postnatal period. Assuming 800 mL daily intake of breast milk, the concentration we observed in our sample would represent approximately 5 mg/day fructose consumption—an amount roughly equal to 1 mg per kg of body weight for a one-month old infant. We recognize that this amount of fructose is very low and far outside the range where fructose is currently known to have physiological effects. Although very small, this concentration could have meaningful effects in developing infants. For example, fructose may be obesogenic in low concentrations in infants where there can be increased susceptibility to chemicals in the environment, including those delivered indirectly as a result of maternal transmission [29,30,31]. There is some evidence that fructose may induce obesogenic effects at very low levels of concentration similar to what we have detected in human breastmilk. For example, in a dose-response study, fructose was shown to increase adipogenesis and induce gene expression in cultured pre-adipocytes, with effects seen at levels as low as 55 µM [32]. This concentration is equivalent to 10 µg/mL, which is only slightly above the concentrations in breast milk that we observed. Of particular significance, even the lowest fructose concentration (10 µg/mL) led to a highly significant and four-fold increase in GluT4 expression in pre-adipocytes, a marker of adipogenesis [32]. Collectively, this study supports the idea that even very low levels of fructose could potentially prime pre-adipocytes towards an adipogenic fate.

Unlike glucose, the metabolism of fructose in unregulated by the liver and affects brain development [8]. Studies have shown that increased levels of fructose can contribute to liver fat, resulting in insulin resistance as well as alterations in insulin and glucose metabolism. However, the dose-response of these effects are completely unknown in infants. The first year of life is a critical developmental period, where even small levels of fructose may have detrimental effects on infant metabolism. In addition, it is possible that the very low levels of fructose reported here are indicating “basal” levels, and higher levels of fructose might be delivered via breast milk to the infant when the mother is consuming sugars during the time of feeding. It is also possible that the detected fructose levels are serving as a proxy measure for some other factor affecting infant growth. More studies are needed to examine the pharmacokinetics of fructose transmission through breast milk in response to maternal consumption. Unfortunately, dietary data were not collected as part of this study; thus, it is not possible to formally determine whether mothers’ habitual consumption of fructose was positively associated with the level of fructose detected in their breast milk. Future studies examining the relationship between maternal diet and breast milk sugar concentrations will be important for informing guidelines for monitoring sugar and fructose intake during the lactation period, and for determining how the Western diet typically affects breast milk composition and infant growth.

Previous studies have shown that higher breast milk glucose concentrations were associated with greater adiposity in infants [12,20]. In the current study, breast milk glucose levels were not related to infant measures of adiposity. It is possible that this discrepancy may be explained by different study designs, or interactions with maternal factors that were not present in our cohort (i.e., gestational diabetes). Ultimately, more studies are needed to determine whether the effects of breast milk sugars are robust and replicable across a variety of infant cohorts and future studies should consider the potential effects of both fructose and glucose in breast milk.

Limitations of the present study include the small sample size in this proof-of-concept study, limited length of follow-up (six months of age), and a lack of dietary intake data that could help explain the relationship between fructose and breast milk sugar composition. Although women were instructed to exclusively breastfeed, it is possible that food introduction may have occurred in some infants during the study period and contributed to growth and body composition, particularly if infants were given access to fructose-containing food products. Indeed, a recent study which evaluated the sugar content of commercial infant and toddler food products found that between 30% and 50% of snacks, desserts, and juices/drinks targeted at infants contain at least one added sugar, with high fructose corn syrup being present in 2%–4% of these items [33]. Many of these items contain sugars in amounts that differ from nutrition labels and often in excess of recommended daily levels [34]. Together, these findings indicate that it is highly likely that infants are exposed to fructose in the first few months of life (e.g., during breastfeeding and/or weaning when complementary foods are introduced to the diet), highlighting the need for further research into the effects of these sugars on child development. Another potential limitation is that we have limited metabolic variables in the mothers. Although we did exclude mothers with type 1 or 2 diabetes based on standard clinical criteria, we were unable to assess prediabetes in this sample. Therefore, it may be possible that mothers with prediabetes had increased levels of insulin and sugars in breast milk. However, breast milk levels of insulin and glucose were not associated with infant growth and body composition.

## 5. Conclusions

Overall, this study suggests a novel mechanism by which infants may be inadvertently exposed to fructose through breast milk, before sugar sweetened beverages and other fructose-containing foods are introduced to the infant diet. This work also opens the door for interventions aimed towards decreased consumption of added sugars while lactating. Future work should be performed with larger samples with longer follow-up (>6 months) in order to establish whether the relationships observed between fructose exposure and infant growth meaningfully impact the development of obesity phenotypes in later childhood and to investigative the mechanism of such an effect at very low levels of fructose. In conclusion, we provide preliminary evidence that fructose is present in breast milk and may be transmitted to the infant, impacting growth and body composition by 6 months of age.

## Figures and Tables

**Table 1:** Participant characteristics.

	1 Month	6 Months	Change ^a^
**Mother**			
Age (years)	29.64 (4.87)		
BMI (kg/m^2^)	28.14 (7.32)		
**Infant**			
Age (days)	39.28 (3.51)		
Sex (F/M)	8/17		
Weight (g)	4766.36 (765.45)	7250.84 (1243.35)	2484.48 (736.89) ^‡^
Length (cm)	55.82 (2.44)	65.38 (2.92)	9.56 (2.07) ^‡^
Lean Mass (g) ^b^	3753.32 (476.02)	4880.05 (749.21)	1126.72 (494.44) ^‡^
Fat Mass (g)	1222.84 (309.11)	2485.08 (642.27)	1262.24 (462.29) ^‡^
Adiposity (%) ^c^	23.96 (3.02)	32.74 (3.75)	8.79 (3.26) ^‡^
BMC (g)	93.2 (17.85)	134.45 (21.0)	41.28 (19.92) ^‡^
BMD (g/cm^2^)	0.37 (0.04)	0.36 (0.03)	−0.002 (0.03)

Mean (SD) for the 25 mother-infant pairs included in this study. ^a^ Significance assessed with paired *t*-tests between 1 and 6 months; ^b^ Lean mass reflects fat-free mass minus BMC (lean tissue only, excluding bone mass); ^c^ Adiposity calculated as percent of fat mass (g) to total mass (g). SD, standard deviation; BMI, body mass index; F/M, females/male; BMC, bone mineral content; BMD, bone mineral density. * *p* < 0.05, ^‡^ *p* < 0.001.

**Table 2:** Changes in milk composition.

	1 Month	6 Month ^a^	Average ^b^
Insulin (pg/mL) ^c^	641.0 (638.0)	640.8 (533.7)	640.9 (469.5)
Fructose (μg/mL)	7.2 (1.72)	6.3 (1.7)	6.7 (1.3)
Glucose (μg/mL)	263.6 (87.5)	246.8 (76.8)	255.2 (75.3)
Lactose (g/dL) ^d^	7.8 (0.8)	7.5 (0.7)	7.6 (0.6)

Mean (SD) for 25 mothers included in this study. ^a^ Change from baseline assessed with paired *t*-test at * *p* < 0.05. All were NS; ^b^ The average value of each sugar (between 1 and 6 months) was used in all statistical models reported; ^c^ One participant excluded for extreme hyperinsulinemia; *n* = 24; ^d^ Data represented in g/dL due to its high concentration in milk.

**Table 3:** Relationships between breast milk sugars and infant body composition at 6 months of age ^a^.

Infant Outcome	Model	β	Δ*R*^2^	Δ*R*^2^ *p* Value
Weight (g)	Base Model		0.70	0.00
	Fructose	256.9 *	0.08	0.02
	Lactose	26.2	<0.01	0.92
	Glucose	−0.9	<0.01	0.73
	Insulin	0.04	<0.01	0.91
Length (cm)	Base Model		0.56	0.00
	Fructose	0.4	0.03	0.28
	Lactose	−1.1	0.04	0.15
	Glucose	−0.01	0.03	0.23
	Insulin	<0.01	0.03	0.21
Weight-for-Length z-score	Base Model		0.52	0.00
	Fructose	0.3 *	0.12	0.02
	Lactose	0.5	0.05	0.14
	Glucose	<0.01	0.01	0.54
	Insulin	<0.01	0.02	0.39
Lean Mass (g) ^b^	Base Model		0.66	0.00
	Fructose	170.1 *	0.09	0.01
	Lactose	224.3	0.03	0.18
	Glucose	−1.8	0.02	0.27
	Insulin	0.30	0.03	0.23
Fat Mass (g)	Base Model		0.59	0.00
	Fructose	130.8 *	0.07	0.05
	Lactose	−31.7	0.00	0.84
	Glucose	−0.2	<0.01	0.88
	Insulin	−0.13	0.01	0.51
Adiposity (%) ^c^	Base Model		0.35	0.03
	Fructose	0.5	0.04	0.29
	Lactose	−1.5	0.05	0.20
	Glucose	0.01	0.01	0.56
	Insulin	<0.01	0.09	0.10
BMC (g)	Base Model		0.59	0.00
	Fructose	4.7 *	0.09	0.03
	Lactose	<0.01	0.01	0.57
	Glucose	<0.01	<0.01	0.76
	Insulin	<0.01	<0.01	0.71
BMD (g/cm^2^)	Base Model		0.47	0.00
	Fructose	<0.01	0.03	0.32
	Lactose	<0.01	0.01	0.62
	Glucose	<0.01	0.07	0.09
	Insulin	<0.01	0.01	0.60

Hierarchical linear regression was used to examine the change in *R*^2^ with the addition of each sugar. The base model included infant sex, 1-month infant weight, and maternal BMI. *R*^2^ is reported for the base model and the change in *R*^2^ is reported in response to adding each sugar to the base model. ^a^ Insulin analyses exclude one participant who was extremely hyperinsulinemic; *n* = 24; ^b^ Lean mass reflects fat-free mass minus BMC (lean tissue only, excluding bone mass); ^c^ Adiposity calculated as percent of fat mass (g) to total mass (g). β, unstandardized regression coefficient; BMC, bone mineral content; BMD, bone mineral density.* *p* <0.05.

## Supplementary Materials

The following are available online at http://www.mdpi.com/2072-6643/9/2/146/s1, Figure S1: Fructose Derivatization Scheme with Methoxyamine (NH_2_O-Me) and *N*-methyl-*N*-(trimethylsily)-trifluroacetamide (MSTFA) for Gas Chromatography-Mass Spectrometry (GC-MS).
