# Supplementary material for: Fructose in Breast Milk Is Positively Associated with Infant Body Composition at 6 Months of Age
Source: Nutrients. 2017 Feb 16;9(2):146. doi: 10.3390/nu9020146 (PMC5331577; doi:10.3390/nu9020146)
Supplement: Supplementary file 1 [file nutrients-09-00146-s001.docx]

Supplementary Materials: Fructose in Breast Milk is Positively Associated with Infant Body Composition at 6 Months of Age

Michael I. Goran, Ashley A. Martin, Tanya A. Alderete, Hideji Fujiwara and David A. Fields

Comparison of GC-MS (Gass Chromatography-Mass Spectrometry) Versus LC-MS/MS (Liquid Chromatography-Mass Spectrometry/Mass Spectrometry)**.** Fructose analysis by GC-MS (Gass Chromatography-Mass Spectrometry) is always risky and hard since fructose is not volatile. Fructose has to be devitalized to become volatile. P. Scano et al. in 2016 Food Research International [1] described the fructose analysis in breast milk (BM) and commercial food (FM) milk by GC-MS. Fructose extract in BM and FM was initially derivatized with methoxyamine HC for 17 h to prepare a fructose methoxyamine derivative followed by silyation, reacted with *N*-methyl-*N*-(trimethylsily)-trifluroacetamide (MSTFA) for 1 h at room temperature. The complete silyation of 4 hydroxy moieties in fructose in the presence of large amounts of other saccharides as well as in the presence of amino acids is always very difficult. After the silyation of fructose, then author’s group added 1.6 µg of tetra deuterated succinic acid d_4_ as the internal standard for fructose quantification. At this point we are not sure that entire 1.6 µg of succinic acid *d*_4_ in the milk sample was completely derivatized (please see attached figure) before GC-MS analysis.

We also believe that the choice of the internal standard of succinic acid *d*_4_ was not suited to fructose quantification since chromatographic properties and MS sensitivities of derivatized fructose and succinate are quite different each other. Furthermore, their GC-MS analyses took more than a day (at least 34 h for 45 milk samples). Thus, some of the silyated compounds (derivatives) might be degraded in the presence of moisture in air during this long analysis time. These problems once happened during the GC-MS analysis, the quantitation data can be easily overestimated.

If the derivation of succinic acid d_4_ was not complete or degradation of derivatized succinic acid *d*_4_ was more than derivatized fructose, the fructose quantification data were overestimated (see equation below).

| Fructose amount (µg/mL) in milk = (GC area of derivatized fructose) × 1.6 µg × (calibration slope)/(GC area of derivatized succinic acid *d*_4_) |  |
| --- | --- |

Fortunately, our HPLC-MS/MS (Liquid Chromatography-Mass Spectrometry/Mass Spectrometry) method on fructose and lactose in breast milk does not require derivatization of fructose and lactose at all. Fructose and lactose are very stable during the analysis. We also used ^13^C_6_ (Carbon-13) labeled fructose as the internal standard. The chromatographic properties and MS sensitivities of both ^13^C_6_ labeled and regular fructose are identical. Thus, we should not have any these issues.

**Figure S1.** Fructose Derivatization Scheme with Methoxyamine (NH_2_O-Me) and *N*-methyl-*N*-(trimethylsily)-trifluroacetamide (MSTFA) for Gas Chromatography-Mass Spectrometry (GC-MS).

Reference

1. Scano, P.; Murgia, A.; Demuru, M.; Consonni, R.; Caboni, P. Metabolite profiles of formula milk compared to breast milk. *Food Res. Int.* **2016**, *87*, 76–82.
